# Supplementary material for: Functional ultrasound imaging of the spreading activity following optogenetic stimulation of the rat visual cortex
Source: Sci Rep. 2021 Jun 15;11:12603. doi: 10.1038/s41598-021-91972-z (PMC8206208; doi:10.1038/s41598-021-91972-z)
Supplement: Supplementary file 1 — Supplementary Information. [file 41598_2021_91972_MOESM1_ESM.pdf]

Functional ultrasound imaging of the spreading activity following optogenetic stimulation of the rat visual cortex

Authors: M. Provansal, G. Labernede, C. Joffrois, A. Rizkallah, R. Goulet, M. Valet, W. Deschamps, U. Ferrari, A. Chaffiol, D. Dalkara, J.A. Sahel, M. Tanter, S. Picaud, G. Gauvain*, F. Arcizet*.

- * Equal contribution

Supplementary informations

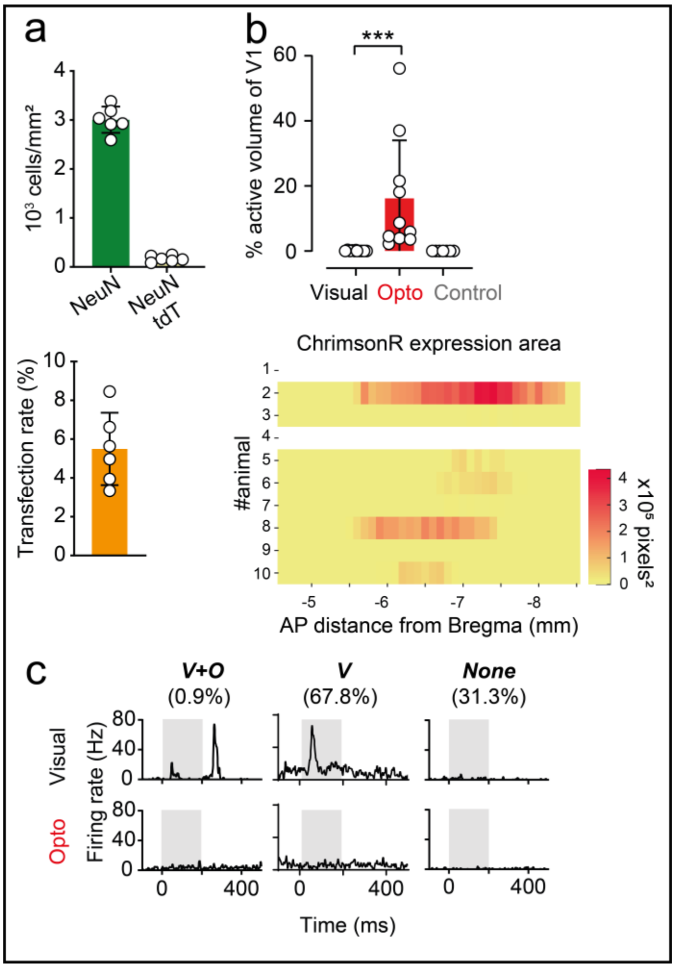

Figure S1, related to Figure 1. (a) Top: NeuN-positive and NeuN/tdT double-positive cell densities. Bottom: neuronal transfection rates. Data were obtained from the brain section displaying the highest tdT fluorescence (*n*=6 animals) (b) Top: percentage active volume of V1 after visual, optogenetic or control stimulation for each animal. For both visual and optogenetic stimulation, we show the volumes of the ipsilateral V1, whereas, for the control, the volume of the contralateral V1 area is shown. Open circles represent values corresponding to single imaging planes, and bars represent the mean (*n*=10 animals, *n*=7 for control, Wilcoxon signed-rank test, one-tailed, *p*=0.001). Bottom: distribution of ChrimsonR expression along the AP axis. (c) Spike density function (SDF) of a typical V1 single units (*n*=118) during visual (top row) or optogenetic (bottom row) stimulation, and from non-injected control animals (*n*=3). Single units were classified into the same subpopulations described in Figure 1.

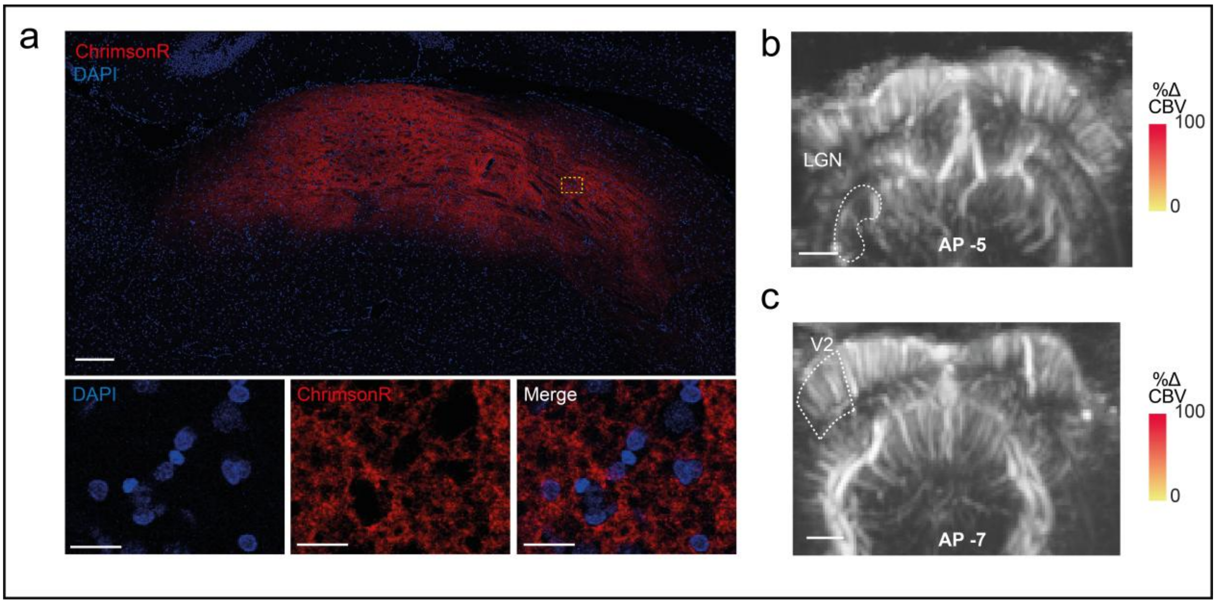

Figure S2, related to Figures 3-4. (a) Top: 20x confocal imaging of a brain section showing ChrimsonR (red) expression in the LGN. Blue: DAPI. Scale bar: 200 µm. Bottom: close-up (40x) of the area delimited by the yellow lines in the previous image. ChrimsonR expression could not be superimposed over DAPI staining. ChrimsonR was, therefore, not present in cell bodies, but was restricted to fibers and dendrites. Scale bar: 20 µm. (b) fUS activation map obtained during control stimulation of the contralateral V1 area from a single imaging plane containing the LGN (AP -5 mm) from the same animal as shown in Figure 3. White dashed lines delimit the contralateral LGN. Scale bar: 2 mm. (c) fUS activation map obtained during control stimulation of the contralateral V1 area from a single imaging plane containing V2 (AP -7 mm) from the same animal as shown in Figure 4. White dashed lines delimit the contralateral V2 area. Scale bar: 2 mm.
